# Extracellular Vesicles from Human Induced Pluripotent Stem Cells Reduce Lung Ischemia-Reperfusion Injury via Cytoprotective Mechanisms in both 2D Cultures and a Human Airway Organoid Model

**DOI:** 10.7150/ijbs.134686

**Published:** 2026-10-02

**Authors:** Rosaria Tinnirello, Maria Chiara Iachini, Maura Cimino, Noemi Elia, Margot Lo Pinto, Simone Dario Scilabra, Gioacchin Iannolo, Giovanni Zito, Roberta Vazzana, Nicola Cuscino, Matteo Bulati, Tuğba Dursun Usal, Federica Cosentino, Antonio D'amore, Federica Guffanti, Giovanna Damia, Vincenza Dolo, Massimo Pinzani, Pier Giulio Conaldi, Alessandro Bertani, Lorenzo Rosso, Lorenza Lazzari, Vitale Miceli

**Affiliations:** 1 Research Department, IRCCS ISMETT (Istituto Mediterraneo per i Trapianti e Terapie ad alta specializzazione), Palermo, Italy.; 2 Unit of Cell and Gene Therapies, Fondazione IRCCS Ca' Granda Ospedale Maggiore Policlinico, Milan, Italy.; 3 Ri.MED Foundation, Palermo, Italy.; 4 Departments of Surgery and Bioengineering, McGowan Institute, University of Pittsburgh, Pittsburgh, USA.; 5 MePreCC, Università degli studi di Palermo, Palermo, Italy.; 6 Università degli Studi di Enna "Kore", Enna, Italy.; 7 Laboratory of Gynecological Preclinical Oncology, Experimental Oncology Department, Istituto di Ricerche Farmacologiche Mario Negri IRCCS, Milan, Italy.; 8 Dipartimento di Medicina Clinica, Sanità Pubblica, Scienze della Vita e dell'Ambiente, Università de L'Aquila, L'Aquila, Italy.; 9 Unit of Thoracic Surgery and Lung Transplantation, IRCCS ISMETT (Istituto Mediterraneo per i Trapianti e Terapie ad alta specializzazione), Palermo, Italy.; 10 Unit of Thoracic Surgery and Transplantation, Fondazione IRCCS Ca' Granda Ospedale Maggiore Policlinico, Milan, Italy.; 11 Department of Medical-Surgical Physiopathology and Transplantation, University of Milan, Milan, Italy.

**Keywords:** lung transplantation, ischemia-reperfusion injury, 3D models, organoids, extracellular vesicles, iPSC.

## Abstract

Lung transplantation (LTx) remains the only curative option for patients with end-stage pulmonary diseases. However, long-term outcomes are compromised by ischemia-reperfusion injury (IRI), a major contributor to primary graft dysfunction and chronic lung allograft impairment. Current pharmacological and technological strategies provide only partial protection, highlighting the need for novel therapeutic approaches. In this study, we investigated the therapeutic potential of extracellular vesicles derived from human induced pluripotent stem cells (hiPSC-EV) in a two-dimensional (2D) *in vitro* model of pulmonary IRI using A549 lung epithelial cells. To better recapitulate the *in vivo* airway microenvironment, we also employed a human airway organoid (hAO) model. Proteomic analysis of hiPSC-EV identified 1,698 proteins enriched in pathways relevant to apoptosis regulation, oxidative stress responses, and inflammatory signaling. In the 2D IRI model, the administration of hiPSC-EV during reperfusion significantly improved cell viability, reduced lactate dehydrogenase (LDH) release, attenuated reactive oxygen species (ROS) accumulation, and decreased apoptosis and necrosis. Furthermore, hiPSC-EV downregulated pro-inflammatory mediators (NFkB1, TNFα, IL1β, IL8, MCP1) while upregulating anti-inflammatory and anti-apoptotic key factors (IL1RA, BCL2). In the 3D model, hiPSC-EV administration preserved organoid structure and viability under IRI conditions, attenuated transcriptional and protein-level expression of pro-inflammatory and oxidative stress markers (IL1β, IL8, HMGB1, HMOX1), and enhanced anti-inflammatory (IL10) and cytoprotective (HSP27, HSP70) responses. Immunofluorescence further revealed that hiPSC-EV reduced activation of apoptotic and pyroptotic pathways. Moreover, intravenous administration of hiPSC-EV in mice showed no adverse safety signals, including tumorigenicity. Together, these findings demonstrate that hiPSC-EV exert broad cytoprotective effects in pulmonary IRI through coordinated regulation of inflammation, oxidative stress, and cell death. Their scalability, reproducibility, and favorable safety profile underscore their promise as innovative cell-free therapeutics to improve outcomes in LTx.

## Introduction

Lung transplantation (LTx) is the gold standard therapeutic option for patients with end-stage pulmonary diseases, offering the potential for significantly prolonged survival and improved quality of life 1. Despite significant advances in surgical techniques, donor organ management and post-operative care, long-term outcomes for lung transplant recipients remain limited due to chronic lung allograft dysfunction (CLAD) and require further improvements 2. Among post-operative transplant complications, one of the most persistent challenges is the ischemia-reperfusion injury (IRI) mechanism, which contributes to primary graft dysfunction (PGD) and early post-operative mortality 3, 4.

IRI is an inevitable consequence of the transplantation process, occurring when the blood supply to the donor organ is interrupted during procurement and subsequently restored upon implantation 5, 6. This condition triggers a cascade of pathological events, including oxidative stress, endothelial dysfunction, and a robust inflammatory response, ultimately leading to tissue injury and impaired graft function 7. In particular, during the ischemic phase, anti-oxidant reservoirs are depleted, and upon reperfusion, rapid reoxygenation further induces tissue damage due to the accumulation of reactive oxygen species (ROS). ROS-induced oxidative stress contributes to extensive cellular damage and local inflammatory responses, leading to a secondary injury via apoptosis, necrosis, and necroptosis 6. Such severe conditions can be associated with high mortality rates in lung transplant patients 8.

Current strategies to mitigate IRI during LTx include ischemic preconditioning 9, optimization of preservation solutions 10, and the use of *ex vivo* lung perfusion (EVLP) systems 11. EVLP, in particular, has emerged as a promising technique, allowing for the assessment and reconditioning of donor lungs prior to transplantation. Moreover, pharmaceutical approaches involving administration of anti-oxidants, anesthetics, and anti-inflammatory medications have been explored 12. However, despite these advancements, effective therapies to prevent or treat IRI remain limited, necessitating the exploration of novel therapeutic approaches.

In this context, mesenchymal stromal/stem cell (MSC)-based therapies have emerged as highly promising approaches for tissue repair and regeneration, due to their ability to modulate inflammation, protect against oxidative damage, and promote healing of injured tissues 13-17. Though originally believed to act via direct engraftment and differentiation, increasing evidence indicates that the primary MSC mechanism of action is paracrine. MSCs secrete a complex mixture of bioactive factors, including cytokines, chemokines, growth factors, and extracellular vesicles (EV), which are collectively referred to as the MSC secretome 18-20.

Among these, MSC-derived EV (MSC-EV) have gained particular attention for their role in mediating many of the beneficial effects of MSCs through cell-to-cell communication 21-23. These nanosized, membrane-bound vesicles carry proteins, lipids, mRNAs, and microRNAs (miRNAs) that can modulate oxidative stress, inflammation, and cell survival. As cell-free entities, EV retain much of the therapeutic potential of MSCs while overcoming several limitations associated with living cell transplantation.

Despite their promise, the clinical translation of MSC therapies has been limited by significant challenges. As primary cells, MSCs are intrinsically difficult to standardize: their phenotype and functional capacity can vary based on donor characteristics, tissue source, and culture conditions. Moreover, MSCs have limited proliferative potential, and tend to undergo senescence during *in vitro* expansion, which reduces therapeutic efficacy. Though MSCs are the most common cell type currently employed in regenerative medicine, their inherent limitations inevitably extend to MSC-EV as well.

In 2007, a groundbreaking technology developed by Yamanaka et al. opened new biological and clinical possibilities by enabling the generation of pluripotent stem cells 24. Using this approach, some of the authors of this paper successfully generated human-induced pluripotent stem cells (hiPSCs) starting from a fetal mesenchymal source, umbilical cord blood, which is recognized internationally as a source of both MSCs and hematopoietic stem cells 25, 26. This allowed researchers to establish a scalable and consistent platform for EV production. Recently, we demonstrated that these hiPSC-EV contain unique mRNA and circRNA cargo, and are capable of promoting tissue regeneration in a model of ischemic brain injury 27. Similarly, other studies have shown comparable effects: for example, Lu et al. reported that hiPSC-EV significantly reduce infarct size, and improve functional outcomes in a rodent model of cerebral ischemia 28.

In this study, we investigated the therapeutic potential of hiPSC-EV in two different *in vitro* models of IRI. To establish a proof-of-concept framework, we first employed a conventional two-dimensional (2D) *in vitro* model to reproduce pulmonary IRI and optimize effective hiPSC-EV treatment conditions, and subsequently validated these findings in a more physiologically relevant 3D human airway organoid (hAO) platform that better recapitulates the structural and cellular complexity of the human airway microenvironment. This 3D model closely recapitulates, at least in part, the native airway tissue, thereby enabling more accurate investigations of the mechanisms underlying lung injury, as well as more reliable preclinical evaluation of candidate therapeutic interventions in a controlled experimental setting. We evaluated the capacity of hiPSC-EV to attenuate oxidative stress, modulate inflammatory responses, and reduce cell death pathways, key hallmarks of pulmonary IRI. Through this investigation, we aimed to provide valuable insights into the development of innovative therapeutic strategies to improve outcomes in LTx.

## Material and Methods

### Culture of human induced pluripotent stem cells

Following the previously described procedures 25, hiPSCs (n=3) were generated from cord blood MSCs. The hiPSC cultures were maintained in StemMACS iPS-Brew XF PSC medium (Miltenyi Biotec, Germany). For standard hiPSC culture maintenance, cells were incubated with 5 mM ethylenediaminetetraacetic acid disodium salt (EDTA; Sigma-Aldrich, USA) in DPBS without Ca^2+^ and Mg^2+^ (Euroclone, Italy), and seeded as cell clumps on hESC-qualified Matrigel Matrix-coated culture plates (Corning, USA). Cells were incubated with Accutase (Biowest, France) and seeded as single cells in the presence of Y-27632 RHO/ROCK pathway inhibitor (Stem Cell Technologies, Canada) at a density of 5,000 cells/cm^2^ on Truncated Vitronectin Recombinant Human Protein-coated culture surfaces (VTN-N; Thermo Fisher Scientific, USA) for hiPSC-EV production, as previously reported 27.

### Isolation and nanoparticle tracking analysis of extracellular vesicles

Cell culture supernatants were collected for hiPSC-EV isolation once the cells reached 70-80% confluence. During the EV collection phase, cells were maintained in StemMACS iPS-Brew XF PSC medium (Miltenyi Biotec, Germany). Conditioned media were collected every 24h for a total of two consecutive collection rounds and pooled for EV isolation.

Cell supernatants were processed through serial centrifugation, as previously described 25, 29. Briefly, cell culture supernatants were pooled, centrifuged at 350 ×g for 10 minutes at room temperature (RT), and further centrifuged at 4,700 ×g for 15 minutes at RT. The resulting cleared supernatants were 0.2 μm filter-sterilized and ultracentrifuged twice at 100,000 ×g for 1 hour at 4 °C using a Sorvall WX 80+ ultracentrifuge equipped with Fiberlite F37L-8×100 fixed angle rotor (Thermo Fisher Scientific, USA). The hiPSC-EV-containing pellets were resuspended and successively washed with 0.1 μm tri-filtered DPBS. The supernatant was discarded, and the obtained ultracentrifuged small hiPSC-EV pellet was resuspended in a total volume of 200 μL.

Samples were assessed by nanoparticle tracking analysis (NTA) using a NanoSight NS300 (Malvern, UK) equipped with a low-volume flow-cell chamber in flow mode, with 5 recordings of 60 seconds each to ensure a constant sample flow and reliable measurements. NTA was performed on each independent hiPSC-EV preparation following isolation. For all functional and characterization experiments, including the ischemia-reperfusion model, we tested the three independent hiPSC-EV batches derived from three hiPSC-EV clones obtained from the same cord blood donor.

### MACSPlex assay

As previously described 29, EV immunophenotyping was performed with the Human MACSPlex exosome kit (130-108-813; Miltenyi Biotec, Germany), following the manufacturer's instructions. Briefly, 120 μL of sample was incubated overnight on a tube rotating mixer at RT in the dark with antibody-coated MACSPlex Exosome Capture Beads. After a washing step with MACSPlex Buffer, the samples were incubated for 1 hour on a tube rotating mixer at RT in the dark with a MACSPlex exosome detection reagent cocktail, comprising APC-conjugated antibodies against CD9, CD63, and CD81. The formation of complexes between antigen-specific capture beads, EV, and the detection reagent was evaluated on a BD FACS Lyric (BD, USA). Each bead population was analyzed for the APC signal to determine antigen expression compared to appropriate isotype control. APC fluorescence values for each marker were normalized to the mean APC intensity of the three tetraspanins as internal reference controls.

### Scanning electron microscopy

HiPSCs were cultured on a glass coverslip using standard culture protocols, fixed in 2% glutaraldehyde (G5882; Sigma-Aldrich, USA) diluted in DPBS (Euroclone, Italy), washed twice with DPBS, and stored at 4 °C. The day of the analysis, the samples were dehydrated through a graded series of ethanol solutions. Samples were critical-point dried and sputter coated with a SCD040 Balzers Sputterer (Balzers Union, Liechtenstein). A Philips 505 SEM microscope (Philips, Netherlands) was used to examine the samples, using an accelerating voltage of 20 kV.

### Mass spectrometry (MS)-based analysis of extracellular vesicles

Ten µg of proteins from three independent hiPSC-EV batches were processed using filter-aided sample preparation (FASP) with 10 kDa Vivacon 500 spin filters 30. Proteins were reduced with 20 mM DTT for 30 minutes at 37°C and alkylated with 50 mM iodoacetamide for 5 minutes at 37°C in the dark (UA buffer: 100 mM Tris-HCl, 8 M urea, pH 8.5) (Sigma Aldrich, USA). After three washes with UB buffer (100 mM Tris-HCl, 8 M urea, pH 8), samples were digested with 0.3 µg LysC (Promega, USA) in UC buffer (25 mM Tris-HCl, 2 M urea, pH 8) for 16 hours at 37 °C, followed by 0.15 µg trypsin digestion in 50 mM ammonium bicarbonate for 4 hours 31. Peptides were eluted, acidified with 0.1% formic acid (Sigma Aldrich, USA), and desalted via STAGE tips (Empore, USA) 32. After elution with 60% acetonitrile/0.1% formic acid (Sigma Aldrich, USA) and vacuum drying, peptides were resuspended in 20 µL of 0.1% formic acid. Peptide concentrations were measured using a Nanodrop 2000 (Thermo Fisher Scientific, USA), and 1 µg of peptides per run was analyzed using a VanquishNeo UHPLC nanoLC system coupled to an Orbitrap Exploris 480 mass spectrometer (Thermo Fisher Scientific, USA) for tandem mass spectrometer analysis. Peptide separation was performed on a PepMap C18 column (25 cm × 75 µm ID) (Thermo Fisher Scientific, USA) with a 131 min gradient. MS data were acquired in data-independent acquisition (DIA) with full MS scans (m/z 400-1000) at 120,000 resolution, AGC target of 3 x 10^6^, and maximum injection time of 50 ms, followed by 60 sequencial DIA windows with an overlap of 1 m/z and windows placement optimization enabled. Raw data were processed using DIA-NN v1.8.0 software against the predicted library generated from *in silico* digested UniProt H. sapiens database UP000005640_9606 (downloaded July 24, 2023), allowing two missed cleavages. Carbamidomethylation (C) was set as a fixed modification; oxidation (M) and N-terminal acetylation were variable. This library consisted of 20,921 protein isoforms, 30,958 protein groups, 6,036,186 precursors in 2,850,551 elution groups. A 1% FDR threshold was applied. Label-free quantification (LFQ) was used for protein quantification and "match between runs" enabled. Data were further analyzed using Perseus 2.0.11 33. The mass spectrometry proteomics data were deposited at the ProteomeXchange Consortium via the PRIDE partner repository, with the dataset identifier PXD068292.

### Cluster analysis and gene ontology (GO) enrichment

Hierarchical cluster analysis of protein abundance (expressed as z-score) was used to group biological and technical replicates showing similar expression patterns. Protein abundance data were grouped using Perseus 2.0.11. To undertake GO enrichment analysis, we analyzed our protein data with the STRING Web Tool 34.

### Human airway organoid generation

Healthy lung biopsies were obtained from young donors undergoing lung resection for spontaneous pneumothorax, in accordance with the ethical standard of the 1975 Declaration of Helsinki. Written informed consent and details of the procedure were approved by the Institutional Research Review Board of the Fondazione IRCCS Ca' Granda Ospedale Maggiore Policlinico, Milan, Italy (project identification code: CE-0006851) and signed by each donor. hAO were generated from tissue-resident adult stem cells and cultured in Cultrex RGF Basement Membrane Extract Type 2 (BME 2) (Bio-Techne, USA), as previously described 35. Every 10-21 days of culture, hAO were removed from BME 2 by cold Dulbecco's phosphate-buffered saline (DPBS) without Ca^2+^ and Mg^2+^ (Thermo Fisher Scientific, USA), enzymatically dissociated with Trypsin-EDTA solution (Thermo Fisher Scientific, USA) for 5 minutes, and cells were then counted with Countess II Automated Cell Counter (Thermo Fisher Scientific, USA), and seeded (1:6-1:12) in fresh BME 2, thus enabling the formation of new organoids. hAO were visualized using an EVOS FL digital inverted fluorescence microscope (Fisher Scientific, UK). Label-free imaging of hAO was also performed using a multiphoton microscopy Leica Stellaris DIVE 8 (Leica Microsystems, Germany), enabling visualization of cellular structures and intrinsic properties while maintaining the native organoid environment. Imaging was conducted using 750 nm excitation, with laser power set between 3-7% and a sampling rate of 40 μs/pixel. Autofluorescence signals were captured via two broad-spectrum emission channels.

### Model of cold ischemia and reperfusion injury in 2D lung cultures and 3D organoids

To simulate the cold ischemia shock and subsequent reperfusion, we used a partially modified protocol, as previously described 15. The ischemia and reperfusion conditions were preliminarily optimized to establish a pulmonary IRI suitable for evaluating the therapeutic effects of hiPSC-EV under conditions of significant cellular stress. Different ischemic durations were initially tested during the model implementation phase. However, milder ischemic insults did not produce sufficiently detectable differences between untreated and hiPSC-EV-treated samples. Therefore, experimental conditions inducing substantial but still partially reversible injury were selected for both 2D and 3D models. In particular, the ischemic phase in the 2D model was achieved by maintaining human alveolar epithelial A549 cells (CCL-185 - ATCC, USA) in Perfadex solution (Xvivo Perfusion, Sweden) for lung preservation at 4 °C for 12 hours (0% O_2_ and 0% CO_2_), followed by simulation of reperfusion obtained by replacing Perfadex solution with complete DMEM (Thermo Fisher Scientific, USA) 10% FBS (Thermo Fisher Scientific, U.S.A.) at 37°C for 5 hours (20% O_2_ and 5% CO_2_). A pre-rewarming step was done by incubating samples at room temperature for 30 minutes. A similar procedure was implemented to mimic IRI in hAO. In this case, however, due to the higher structural and cellular complexity of the organoids, a longer ischemic phase (16 hours) was required to induce a substantial injury while preserving the possibility to evaluate treatment-associated recovery during 24 hours of reperfusion using adDMEM/F12 (Thermo Fisher Scientific, USA) with supplements (10 mM Hepes, 1 × penicillin/streptomycin, 1 × Glutamax, 1% B27, 1.25 mM N-acetylcysteine, 25 ng/mL FGF7, 500 ng/mL RSPO1, 100 ng/mL FGF10, 100 ng/mL Noggin, 5 mM nicotinamide, 500 nM A83.01, and 500 nM SB202190, Thermo Fisher Scientific, USA) as complete medium for reperfusion. hiPSC-EV were administered at the beginning of the reperfusion phase at a concentration of 10,000 particles/cell in both 2D and 3D model. The hiPSC-EV dose used in the experiments was selected based on preliminary dose-response analyses performed in the 2D IRI model. In particular, the dose of 10,000 particles/cell was identified as the most effective concentration in improving cell viability after reperfusion and was subsequently adapted to the 3D organoid model according to the estimated number of cells per well. In particular, the estimated number of cells per organoid was preliminarily determined by dissociating organoids of comparable diameter and performing cell counts.

### Cell death and cell cycle assessment

We analyzed cell viability in both 2D and 3D cultures by quantifying viable cells with CellTiter-Glo 3D Cell Viability Assay, according to the manufacturer's instructions (Promega, USA). Cell death in 2D cultures was analyzed by detection of the release of lactate dehydrogenase (LDH) in cell culture media. Enzyme activity was measured using the Dimension Vista Intelligent Lab System, according to the manufacturer's instructions (Siemens Healthcare Diagnostics, USA). The cell cycle was measured with propidium iodide (PI) staining (50 μg/mL) (Sigma Aldrich, USA). In particular, A549 cells were detached using Trypsin-EDTA solution (Thermo Fisher Scientific, USA), washed once, and resuspended in 300 μL cold DPBS (Thermo Fisher Scientific, USA). The cells were then fixed by adding 700 μL cold pure ethanol (Sigma Aldrich, USA) dropwise during slow vortexing (70% final concentration) and incubated on ice for 30 minutes. Fixed cells were washed once with DPBS and subsequently resuspended in 1 mL PI (50 μg/mL) containing RNase (250 μg/mL) (Sigma Aldrich, USA) for 60 minutes, light-protected, and at room temperature. PI fluorescence was detected using the FACSCelesta cytometer (BD Life Sciences, UK).

### Intracellular reactive oxygen species quantification

Intracellular ROS levels in A549 cells were measured by 2′,7′-Dichlorodihydrofluorescein diacetate (DCFH-DA) (Sigma Aldrich, USA), an organic dye of the fluorescein family that reacts with intracellular radicals to produce the oxidized DCF, a fluorescent product that is retained within the cells 36. The cells were incubated with 10 µM DCFH-DA for 40 minutes at 37 °C, and then washed twice with DPBS (Thermo Fisher Scientific, USA). Fluorescence was detected at an excitation wavelength of 485 nm and an emission wavelength of 530 nm using a FACSCelesta cytometer (BD Life Sciences, UK).

### Gene expression analysis

We performed real-time PCR to analyze mRNA expression with TaqMan assays, according to the manufacturer's instructions (Thermo Fisher Scientific, USA). We examined 7 genes related to inflammation, namely nuclear factor kappa B subunit 1 (NFkB1) (Hs00765730_m1), tumor necrosis factor alpha (TNFα) (Hs00174128_m1), interleukin 1 beta (IL1β) (Hs01555410_m1), interleukin 8 (IL8) (Hs00174103_m1), monocyte chemoattractant protein 1 (MCP1) (Hs00234140_m1), and interleukin 1 receptor antagonist (IL1RA) (Hs00893626_m1); 3 genes related to oxidative stress, namely nuclear factor erythroid 2-related factor 2 (NRF2) (Hs00975961_g1), heme oxygenase 1 (HMOX1) (Hs01110250_m1), and musculoaponeurotic fibrosarcoma oncogene homolog (MAF) (Hs04185012_s1), and the anti-apoptotic gene B-cell lymphoma 2 (BCL2) (Hs04986394_s1). Total RNA was extracted with the miRNeasy Mini Kit and treated with DNAse (QIAGEN, Germany). The purity and quantity of isolated RNA were determined by OD 260/280 using a NanoDrop Spectrophotometer (Thermo Fisher Scientific, USA). Subsequently, 250 ng of RNA were transcribed with the high-capacity RNA-to-cDNA kit protocol (Thermo Fisher Scientific, USA) to produce single-stranded cDNA. We analyzed gene expression with the QuantStudio 7 Pro RealTime PCR System (Thermo Fisher Scientific, USA) using the glyceraldehyde 3-phosphate dehydrogenase (GAPDH) (Hs99999905_m1) as a housekeeping gene. The fold change in mRNA expression was determined according to the 2^-∆∆Ct^ method.

### Protein expression analysis

The abundance of selected proteins related to inflammation, such as IL1β, interleukin 2 (IL2), IL8, interleukin 10 (IL10), high mobility group box 1 (HMGB1), and oxidative stress, such as HMOX, heat shock protein 27 (HSP27), and heat shock protein 70 (HSP70), was measured in the conditioned medium of hAO during IRI. The analysis was done with Luminex magnetic bead technology with the ProcartaPlex Human Luminex Kit (Thermo Fisher Scientific, USA), following the manufacturer's instructions. Proteins were analyzed with the Luminex xMAP INTELLIFLEX System (Thermo Fisher Scientific, USA).

### Immunofluorescence staining and analysis

For immunofluorescence staining, hAO were retrieved from BME2, washed with DPBS, and fixed with 4% paraformaldehyde (Sigma Aldrich, USA) for 30 minutes on ice. Samples were then permeabilized with 0.1% Triton X-100 and 1% bovine serum albumin (BSA, Thermo Fisher Scientific, USA) in DPBS (Thermo Fisher Scientific, USA) for 40 minutes at room temperature (RT). Following permeabilization, hAO were washed with DPBS and blocked for 1 hour at RT in blocking solution containing 1% BSA in DPBS. hAO were then incubated overnight at 4 °C with the following primary antibodies diluted in blocking solution: anti-human mucin 1 (MUC1) (10 µg/ml; Abcam, UK); anti-human secretoglobin (SCGB1A1) (dilution 1:1000; Abcam, UK); anti-human cytokeratin 5 (KRT5) (dilution 1:100; Abcam, UK); anti-human acetylated α-tubulin (AcTub) (dilution 1:100; Sigma Aldrich, USA); anti-human cleaved caspase 3 (cCASP3) (dilution 1:100; Cell Signaling, USA); anti-human cleaved gasdermin D (cGSDMD) (dilution 1:100; Cell Signaling, USA); anti-human phospho mixed lineage kinase domain-like protein (pMLKL) (dilution 1:500; Cell Signaling, USA). After two washes with DPBS, hAO were incubated with goat anti-rabbit or anti-mouse IgG secondary antibody (with Cy3 and fluorescein, respectively; AffiniPure donkey anti-rabbit or anti-mouse IgG, Jackson ImmunoResearch, USA) at a concentration of 10 µg/ml and with 4′,6-diamidino-2-phenylindole dihydrochloride (DAPI) (concentration 1 μg/mL; Sigma-Aldrich, USA) for 1 hour at room temperature. Following two additional washes with DPBS, coverslips were mounted on slides using Vectashield Antifade Mounting medium (VectorLab, USA), and samples were visualized under a Leica confocal station (Leica SP5 confocal system) mounted on a Leica DM6000 inverted microscope (Leica Microsystems Inc., USA). Quantification of immunofluorescence was calculated with ImageJ software v1.54 (NIH, USA).

### Safety of hiPSC-EV in animal models

For safety studies, 10 μg of hiPSC-EV (n=3 batches) in a total volume of 200 μL of DPBS (Euroclone) were administered intravenously in the tail of CD1 nude female mice (CHARLES RIVER Laboratories, Calco, Italy) (n=3 per each hiPSC-EV). The mice were monitored weekly, and body weight was registered across 3 months. Mice were maintained under specific pathogen-free conditions, housed in isolated vented cages, and handled using aseptic procedures. Anesthesia was not used in this investigation. At the end of the experiments, the mice were euthanized using inhaled CO_2_ in controlled increasing doses. This study is reported in accordance with the ARRIVE (Animal Research: Reporting of *In vivo* Experiments) guidelines 2.0.

### Statistical analysis

All data were obtained from at least three independent experiments and are expressed as mean ± SD. Statistical analyses were done using GraphPad Prism 6.0 (GraphPad Software, U.S.A.). For statistical comparison, the one-way ANOVA followed by Tukey's multiple comparison test was used. Differences were considered statistically significant at p < 0.05.

## Results

### hiPSC-EV characterization

Size profile and surface marker composition of hiPSC-EV were assessed to investigate particle features, excluding possible artifacts derived from serial ultracentrifugation. First, to characterize the physical properties of hiPSC-EV, NTA was performed (Fig. 1A). Our analysis showed a homogeneous population of nanoparticles, without multimodal peaks suggesting the absence of significant aggregation. The average size (160-180 nm) was consistent with previously reported hiPSC-EV, defined according to the MISEV guidelines by a size < 200 nm 37, 38. A comprehensive molecular characterization of hiPSC-EV was made to identify the expression of markers related to cell identity, origin and biogenesis pathways. The MACSplex assay, shown in Figure 1B, revealed the presence of EV-enriched tetraspanins, CD9, CD63, and CD81. Markers indicative of pluripotent and multipotent progenitor states (SSEA-4, CD133/1), early embryogenesis (ROR1), and epithelial and stem cell adhesion (CD326, CD29) were found to be highly expressed (Fig. 1B). Conversely, antigens linked to mesenchymal stromal and endothelial cell characterization and function (CD146, CD105, CD56, CD49e, CD44, NG2) and immune system cells (CD41b, CD45, CD31, CD209, CD142,) were not present (Fig. 1B). Remarkably, major histocompatibility complex classes, HLA-ABC and HLA-DR/DP/DQ were not detected in hiPSC-EV. Scanning electron microscopy exhibited a distinct localized blebbing of EV at the edges of hiPSC colonies (Fig. S1). Additional in-depth biochemical and molecular data of hiPSC-EV characterization are reported in our previously published study 27.

### Proteomic profiling of hiPSC-EV

To assess the functional protein cargo of EV, three independent hiPSC-EV batches were analyzed in technical triplicates using mass spectrometry-based proteomics. In total, 1,698 proteins were identified across the samples, with each protein detected in at least two replicates per batch. Hierarchical clustering revealed highly consistent proteomic profiles both among technical replicates and across biological batches, indicating robust reproducibility (Fig. 2A). Functional annotation through GO (biological process) analysis revealed that the EV protein cargo was significantly enriched in pathways relevant to IRI pathogenesis, including regulation of apoptosis, oxidative stress responses, and modulation of inflammatory signaling (Fig. 2B). To further explore the molecular interactions underlying these pathways, protein-protein interaction (PPI) network analysis was done, highlighting key hub proteins potentially mediating the EV therapeutic effects (Fig. 2C). These findings provide a strong rationale for testing hiPSC-EV in our experimental models of pulmonary IRI.

### Therapeutic effects of hiPSC-EV in a 2D model of pulmonary IRI

To evaluate the hiPSC-EV protective effects against pulmonary IRI, we first employed a 2D model using the A549 human cell line. According to the experimental plan detailed in Fig. 3A, cells were grown in standard conditions (control) or subjected to cold ischemia followed by a reperfusion phase to mimic the IRI conditions. We tested the hiPSC-EV therapeutic properties, administered during the reperfusion phase, in order to reduce the side effects of IRI (Fig. 3A).

After 12 hours of ischemia, marked morphological alterations were evident compared with control cells, with the presence of rounded cells (Fig. 3B). Following an additional 5 hours of reperfusion, these changes were exacerbated, with a pronounced reduction in the number of adherent viable cells. Notably, the treatment with hiPSC-EV partially attenuated this effect (Fig. 3B). To confirm these preliminary observations, and to assess whether particle administration during the reperfusion phase exert protective effects against IRI, we evaluated both cell viability and cell death by ATP quantification and LDH release, respectively. As shown in Fig. 3C, cell viability decreased significantly from 100% (control) to 3.26% (± 0.31%) in the IRI group. In contrast, hiPSC-EV-treated samples displayed a viability of 36.01% (± 1.78%). These findings were corroborated by the LDH release assay, which revealed LDH levels of 31 U/L following IRI, and a reduced level of 16 U/L in the IRI + EV-treated group (Fig. 3D).

Indeed, a distinct pattern of cell damage and death was observed depending on hiPSC-EV presence or absence, as demonstrated by cell cycle analysis. Specifically, IRI exposure led to a marked increase in cell cycle arrest, with 37.9% (± 4.51%) of cells accumulating at a G2/M phase, whereas hiPSC-EV treatment reduced the proportion of arrested cells to 28.9% (± 3.83%) (Figs. 4A and 4B). Moreover, a predominant apoptotic/necrotic profile was detected in IRI samples, with 5.2% (± 0.31%) of cells undergoing apoptosis/necrosis, while this percentage decreased to 3.8% (± 0.28%) in the hiPSC-EV-treated group (Figs. 4A and 4C).

To further investigate the mechanisms underlying the hiPSC-EV protective effects against IRI in A549 cells, we assessed the ROS production. Notably, after IRI, a significant increase in ROS levels occurred, 12.5-fold compared to the control. Administration of hiPSC-EV significantly reduced ROS production, bringing it down to a 9.7-fold increase relative to control (Figs. 4D and 4E).

The protective effects observed in the A549 model, following hiPSC-EV treatment, were further supported by molecular biology analyses. Specifically, we examined key regulators of both inflammation and apoptosis processes. Pro-inflammatory factors, including NFkB1, TNFα, IL1β, IL8, and MCP1, were significantly upregulated after 5 hours of IRI (IRI 5h), showing increases of 5.7-, 5.2-, 2-, 1.35-, and 2.7-fold, respectively, compared to the control. hiPSC-EV treatment significantly inhibited the transcriptional activation of these pro-inflammatory mediators. Regarding the anti-inflammatory factor, IL1RA was markedly upregulated by IRI 5h (21.4-fold vs. control), and hiPSC-EV administration further enhanced its expression reaching 25.7-fold compared to the control. Additionally, analysis of BCL2, a key anti-apoptotic factor, revealed a 2.4-fold reduction in expression following IRI 5h, which was restored by the presence of hiPSC-EV (Fig. 5).

### Human airway organoids recapitulate the final portion of the lung airway and provide a relevant model for IRI studies

To extend our investigation to a more physiologically relevant system, we developed a lung organoid-based model (Fig. S2A) to evaluate the effects of hiPSC-EV under IRI conditions. Prior to injury induction, we characterized the organoid cellular composition using immunofluorescence staining for key cell markers. Staining revealed the presence of cells that can be found in the final portion of the lung airway, goblet cells marked by MUC1, ciliated cells recognized by AcTub, club cells identified by secretoglobin expression, and basal cells positive for KRT5 (Fig. S2B), confirming the multicellular identity of the airway organoids. This complex cellular architecture provides a robust platform for assessing hiPSC-EV-mediated cytoprotection in a context that better recapitulates the *in vivo* lung environment.

### Therapeutic effects of hiPSC-EV in a 3D airway organoid IRI model

To assess the therapeutic impact of hiPSC-EV in a 3D airway organoid model of IRI, hAO were subjected to an ischemia and reperfusion protocol, as shown in Fig. 6A. Following 16 hours of cold ischemia and 30 minutes of rewarming, organoids underwent reperfusion with or without hiPSC-EV treatment, and were analyzed after 2, 6, and 24 hours. Bright-field microscopy images revealed substantial morphological changes after 16 hours post-ischemia (black arrows) compared to the control (Fig. 6B). This damage increased (black arrows) after 2, 6, and 24 hours post-reperfusion (Figs. 6C-6E). In contrast, hiPSC-EV-treated organoids preserved their structural integrity over time, showing fewer signs of injury at different post-reperfusion time points (Figs. 6C-6E). This finding was further supported by multiphoton microscopy analysis of hAO (Fig. 6F). Quantitative assessment of viability revealed a marked decrease after 16 hours post-ischemia (Fig. 6G). A significant recovery at all post-reperfusion time points with hiPSC-EV administration (Fig. 6G) was observed, highlighting the protective effects of EV in this 3D tissue model compared to samples untreated during the reperfusion phase.

### hiPSC-EV modulate inflammatory and anti-oxidant gene and protein expression in airway organoids following IRI

To investigate the molecular mechanisms underlying the protective effects of hiPSC-EV, we analyzed the expression of key inflammatory and anti-oxidant genes in airway organoids subjected to IRI with or without hiPSC-EV treatment. Gene expression profiling revealed that IRI induced a significant upregulation of pro-inflammatory genes, including NFkB1, IL1β, and IL8, particularly during early post-reperfusion (2 hours), which was significantly attenuated by hiPSC-EV treatment (Fig. S3, top panel). In contrast, the anti-inflammatory cytokine IL10 was further upregulated in hiPSC-EV-treated hAO compared to untreated counterparts, suggesting an immunomodulatory shift toward resolution of inflammation (Fig. S3, top panel). Additionally, the expression of oxidative stress-related genes was assessed. While NRF2 levels remained stable, expression of MAF and HMOX1 was strongly increased following IRI, with significantly lower expression in the hiPSC-EV-treated group at all post-reperfusion time points (Fig. S3, bottom panel), indicating a possible reduction of oxidative stress.

At the protein level, these transcriptional trends were confirmed. Indeed, a significant increase was observed after ischemia followed by 2 hours of reperfusion for IL1β, IL2, and IL8, while HMGB1 expression increased after 6 hours of reperfusion. Interestingly, their expression was reduced in hAO treated with hiPSC-EV (Fig. 7, top panel), while IL10 protein levels significantly increased after 2 hours hiPSC-EV administration (Fig. 7, top panel), validating the anti-inflammatory effect observed at the gene level (Fig. S3, top panel). Moreover, while the key anti-oxidant and stress response protein HMOX1 was highly abundant after IRI, with substantially lower levels in hiPSC-EV-treated samples across all reperfusion time points, the molecular chaperones HSP27 and HSP70 were more abundant across all reperfusion time points in hiPSC-EV-treated samples (Fig. 7, bottom panel).

These data indicate that hiPSC-EV confer protective effects by modulating both transcriptional and translational responses to IRI, attenuating inflammation and oxidative stress in hAO.

### Multiple death pathways are activated by IRI and reduced by hiPSC-EV

To investigate the cell death mechanisms triggered by IRI in hAO, and to elucidate how hiPSC-EV can confer cytoprotection, immunofluorescence staining for cleaved caspase-3 (cCASP3; apoptosis), cleaved gasdermin D (cGSDMD; pyroptosis), and phospho mixed lineage kinase domain-like protein (pMLKL; necroptosis) was done. hAO were analyzed pre-ischemia, post-ischemia (16 hours), and 24 hours after IRI (Fig. 8).

Staining for cCASP3 showed a significant increase in apoptotic signaling post-ischemia, which was reduced after IRI at 24 hours without hiPSC-EV. Interestingly, hAO after IRI at 24 hours with hiPSC-EV administration exhibited a marked reduction in cCASP3 signal intensity compared to IRI at 24 hours without treatment, indicating a decrease in apoptosis (Fig. 8A).

Moreover, the staining for the key executioner of pyroptosis, cGSDMD, showed an increased signal intensity level post-ischemia, which was steadily maintained after 24 hours of IRI (IRI 24h) and significantly reduced with hiPSC-EV administration (Fig. 8B). pMLKL, a hallmark of necroptosis, was also strongly upregulated post-ischemia, and was markedly reduced after IRI 24h mainly in hiPSC-EV-treated hAO (Fig. 8C). Quantitative analysis confirmed statistically significant reductions in apoptotic, pyroptotic, and necroptotic markers in the hiPSC-EV-treated group compared to IRI 24h untreated (Fig. 8D). These findings indicate that IRI activates multiple, concurrent forms of programmed cell death in airway organoids, and that hiPSC-EV exert a broad cytoprotective effect by attenuating apoptosis, pyroptosis, and necroptosis.

### Safety of hiPSC-EV in animal model

In order to envision a possible clinical use of these hiPSC-EV, we decided to completely rule out the safety issues after having already demonstrated the absence of full-length transcription factors associated with pluripotency 27. hiPSC-EV were intravenously administered to a CD1 nude mouse model. The animals were monitored for 3 months for body weight, general health, and presence of visible teratomas. No abnormalities of any kind were observed (Table 1).

## Discussion

In this study, we evaluated the therapeutic potential of hiPSC-EV in attenuating IRI using human pulmonary *in vitro* models. IRI remains a major barrier to successful LTx, contributing to PGD and CLAD 2-4. Pharmacological treatments and EVLP are being explored as potential strategies to reduce lung IRI, yet their impact remains suboptimal and is often constrained by donor organ heterogeneity and strict procedural timing 12, 14.

MSC-based therapies are among the most widely applied approaches in regenerative medicine and have demonstrated potential in IRI models of various organs, including the heart, liver, kidney, and lung 14-16, 39, 40. Growing evidence indicates that many of these effects are mediated through paracrine mechanisms, particularly EV, which can modulate inflammation, oxidative stress, and apoptosis 21, 41-44. In particular, MSC-EV have previously shown protective effects in several experimental models of IRI through anti-inflammatory, anti-oxidant, and anti-apoptotic mechanisms 45-50. Notably, MSC-EV has also been shown to improve pulmonary energetics and function during EVLP 51. These observations collectively support the broader concept that EV-mediated paracrine signaling may represent a promising cell-free therapeutic strategy also in lung transplantation. Moreover, EV appears advantageous due to their biocompatibility, stability, ease of administration, and low immunogenicity 52, making them attractive therapeutic candidates in IRI.

After focusing our interest on a proteomic analysis of the MSC-derived secretome, demonstrating that EV significantly contribute to MSC-mediated repair by delivering functional protein cargo 21, in this study we turned our attention to a novel and promising EV population with distinct regenerative potential. The reason, as mentioned in the introduction, is that one of the main limitations of considering the use of EV is due to the fact that MSCs are primary cells and therefore difficult to standardize, with donor-dependent variability in their efficacy. This variability is also reflected in the composition and functional properties of their EV 53. The advent of cellular reprogramming has opened new perspectives for EV-based therapeutics, enabling the generation of iPSC as a renewable, standardized, and well-characterized source of EV, thereby overcoming the intrinsic donor-related variability of MSC-derived preparations. Recent studies have increasingly highlighted the therapeutic potential of hiPSC-derived EV as a cell-free platform for regenerative medicine applications. These EV have already demonstrated protective effects in different IRI models, including renal and cardiac injury, through modulation of oxidative stress, inflammation, and apoptosis pathways 54-56.

In line with our previous findings demonstrating the regenerative potential of hiPSC-EV 27, the present study further supports the concept that hiPSC-derived EV may represent a robust and versatile platform for regenerative medicine applications, including pulmonary protection during IRI. Here, unlike studies focusing on RNAs, we emphasized the functional role of the protein cargo of hiPSC-EV. Proteomic analysis identified 1,698 proteins consistently present across biological and technical replicates. Interestingly, the highly consistent proteomic profiles observed across independent hiPSC-EV batches in our study (Fig. 2A) further support a potential advantage of hiPSC-derived EV in terms of reproducibility and reduced donor-associated variability, a limitation frequently reported for MSC-EV preparations 55. GO and PPI analyses revealed enrichment in pathways related to apoptosis regulation, oxidative stress response, and inflammation, supporting their relevance in IRI modulation (Figs. 2B-C). Initial experiments in a 2D A549 cell model of pulmonary IRI demonstrated that hiPSC-EV treatment during reperfusion significantly improved cell viability, reduced LDH release (Fig. 3), and lowered ROS levels (Fig. 4). hiPSC-EV also partially reversed cell cycle arrest and apoptosis/necrosis (Fig. 4). Notably, hiPSC-EV treatment restored BCL2 expression, suppressed pro-inflammatory markers (NFkB1, TNFα, IL1β, IL8, MCP1), and enhanced the anti-inflammatory mediator IL1RA (Fig. 5).

To better mimic the *in vivo* environment, we used an innovative human airway organoid model, not yet explored in the context of IRI. Immunofluorescence confirmed the presence of multiple functional cell types, including goblet, ciliated, club, and basal cells (Fig. S2). hiPSC-EV treatment preserved organoid structure and viability following IRI, in contrast to progressive degeneration in untreated controls (Fig. 6). Gene expression analysis revealed that IRI induced upregulation of pro-inflammatory (NFkB1, IL1β, IL8) and oxidative stress genes (MAF, HMOX1), while hiPSC-EV treatment attenuated these responses and increased IL-10 expression (Fig. S3). Though MAF is not a primary regulator of oxidative stress, it modulates genes involved in cellular redox homeostasis. In parallel, HMOX1, an adaptive stress response protein, is upregulated following ROS elevation 57, 58. hiPSC-EV treatment attenuated these signals, resulting in reduced MAF and HMOX1 expression (Fig. S3). At the protein level, we observed similar reductions for pro-inflammatory factors (IL1β, IL2, IL8, HMGB1) and the stress response protein HMOX1 following hiPSC-EV administration (Fig. 7). Notably, two anti-oxidant heat shock proteins, HSP27 and HSP70, were highly abundant at each reperfusion time point with hiPSC-EV treatment (Fig. 7). Immunofluorescence for cCASP3 (apoptosis), cGSDMD (pyroptosis), and pMLKL (necroptosis) showed that IRI activates multiple cell death pathways. hiPSC-EV treatment significantly attenuated apoptotic, pyroptotic, and necroptotic responses (Fig. 8).

These findings highlight the broad cytoprotective properties of hiPSC-EV, targeting key mechanisms of IRI-induced injury. Our results, which showed reduced HMGB1 and HMOX1 (a key sensor of oxidative stress) 57, and increased IL-10, align with prior *in vivo* studies 59. Furthermore, as revealed in our 2D and 3D study models, hiPSC-EV treatment reduced inflammation, oxidative stress and cell death, consistent with the literature attributing these effects to EV-carried miRNAs 45, 46, 48, 50, 60, 61. Our proteomic data provides additional support to the observed functional effects, revealing an enrichment of hiPSC-EV cargo in pathways implicated in the response to IRI (Figs. 2B and C). In particular, the presence of several key proteins (Table S1) appears to align closely with our experimental findings, obtained in both 2D and 3D airway models post-IRI. These include reduced ROS levels, downregulation of pro-inflammatory mediators, restoration of anti-apoptotic signaling, and modulation of cytokine responses. It is worth noting that hiPSC-EV contained two heat shock proteins, HSP27 (HSPB1) and HSP70 (HSPA1A) (Table S1), both previously associated with cytoprotection and cellular stress resilience, and found to be enriched in hiPSC-EV-treated samples. These proteins inhibit caspase-3 activation, interact with Apaf-1 to block apoptosome formation, and stabilize BCL-2 62-66. Other proteins contained in our hiPSC-EV also possess pleiotropic effects on both apoptosis and oxidative stress pathways, including GSTP1, DNAJA1, PARK7, NQO1, and MIF (Table S1). GSTP1 protects cells during oxidative stress 67. DNAJA1 supports HSP70 function in mitigating apoptosis 68. PARK7 possesses an anti-oxidant function by stabilizing NRF2 function and inhibiting apoptosis 69. NQO1 and MIF inhibit oxidative stress and p53-mediated apoptosis 70-72. Interestingly, hiPSC-EV also contained crucial anti-oxidant factors such as superoxide dismutase 1 and 2 (SOD1 and 2) and catalase (CAT) (Table S1), which neutralize superoxide anions and hydrogen peroxide, respectively 73. Moreover, two stress response and survival proteins, HSP90AA1 and HSPA1A, were also detected in our hiPSC-EV (Table S1), likely contributing to the observed preservation of cell viability and structural integrity 74, 75. Additionally, hiPSC-EV contained immunomodulatory proteins such as SERPINF1, GRN (Granulin), and METRNL (Meteorin-like protein) (Table S1), which may regulate immune responses and cytokine production 76-79. However, our proteomic analysis should be considered hypothesis-generating, providing a prioritized set of candidate mediators that will require dedicated functional validation to establish their direct contribution to the cytoprotective effects of hiPSC-EV.

Although the hAO model used in this study successfully recapitulates several key epithelial features of the distal airway, the lack of mesenchymal, endothelial, and immune cell compartments represents an important limitation of the present study. Consequently, the model does not allow comprehensive investigation of the multicellular crosstalk occurring during IRI *in vivo*. Nevertheless, we deliberately chose to initially focus on an epithelial-based organoid platform because epithelial cells represent one of the primary targets of IRI and because this system offers several experimental advantages, including reproducibility, controlled analysis of epithelial-specific responses, relative ease of culture and manipulation, and the possibility to directly evaluate hiPSC-EV-mediated cytoprotective effects in a human-derived airway context. At the same time, epithelial-mesenchymal, epithelial-endothelial, and immune-mediated interactions are known to critically contribute to inflammatory signaling, tissue remodeling, vascular dysfunction, and tissue repair processes following IRI. Future studies should therefore aim to implement more advanced multicellular and co-culture platforms in order to further validate and better dissect the therapeutic effects and mechanisms of hiPSC-EV in a more physiologically representative setting.

## Conclusion

In summary, our findings highlight the therapeutic promise of hiPSC-EV in mitigating lung IRI through multifaceted mechanisms. By integrating *in vitro* 2D and 3D human airway models with proteomic profiling of hiPSC-EV cargo, we provide a comprehensive overview of their potential cytoprotective actions. The consistent presence of proteins involved in oxidative stress response, apoptosis regulation, and immune modulation, together with the functional improvements observed post-treatment, suggest that hiPSC-EV may act as active mediators of tissue repair. Given their scalability, reproducibility, and donor-independence, hiPSC-EV may overcome critical barriers associated with cell-based therapies. In addition, our safety data from animal models reinforce their potential for clinical translation, while demonstrating the absence of tumorigenic effects typically associated with the parental iPSC population.

Though further studies are needed to validate individual protein functions and optimize delivery strategies, our data lays the foundation for future application of hiPSC-EV in clinical settings, including organ preservation protocols such as EVLP and early post-transplant interventions. These findings support a growing interest in EV-based, cell-free therapies as innovative and adaptable tools to improve outcomes in lung transplantation.

## Supplementary Material

Supplementary figures.

## Figures and Tables

**Figure 1:**
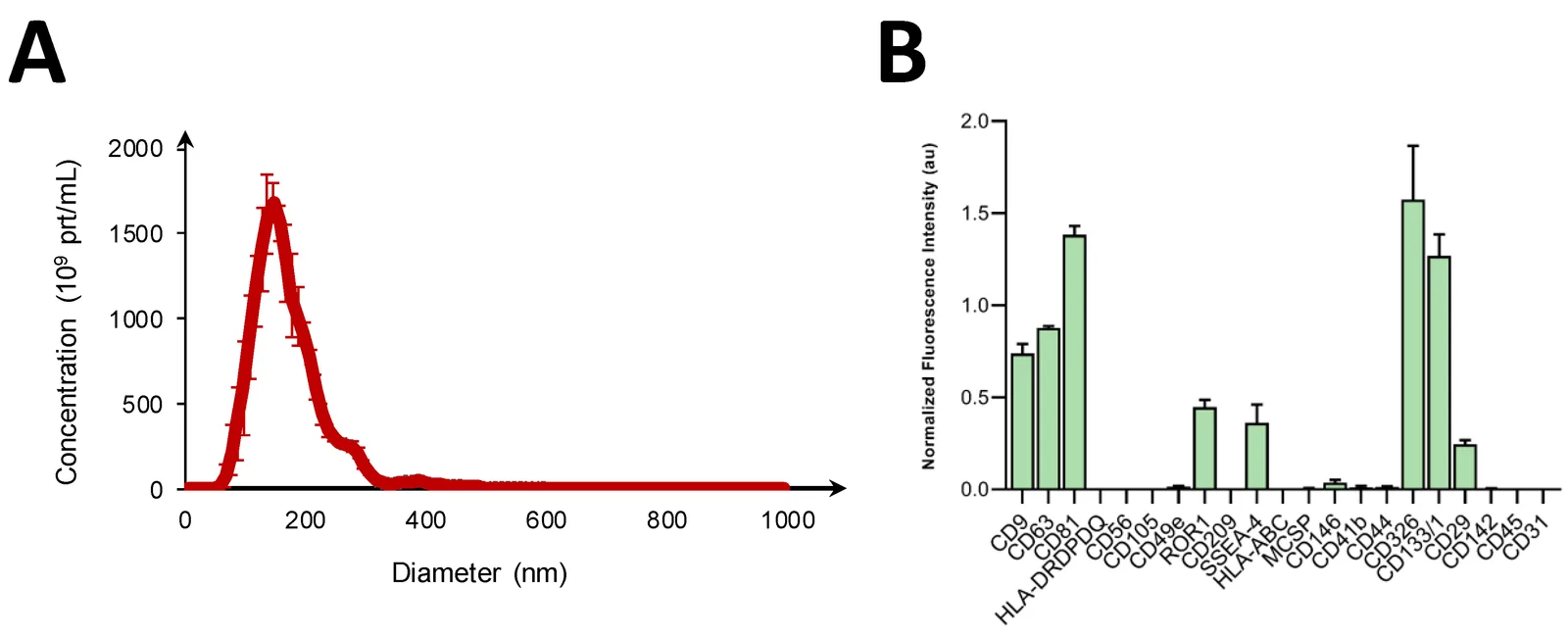
hiPSC-EV characterization. (A) NTA histogram showing size distribution profile of particles released by hiPSCs (n=3). (B) Histograms showing signal intensity (arbitrary units) of surface protein markers on hiPSC-EV (n = 3), as detected by the MACSPlex Exosome Kit and analyzed via flow cytometry. Data reported in the graph are mean ±SEM of n=3 independent experiments.

**Figure 2:**
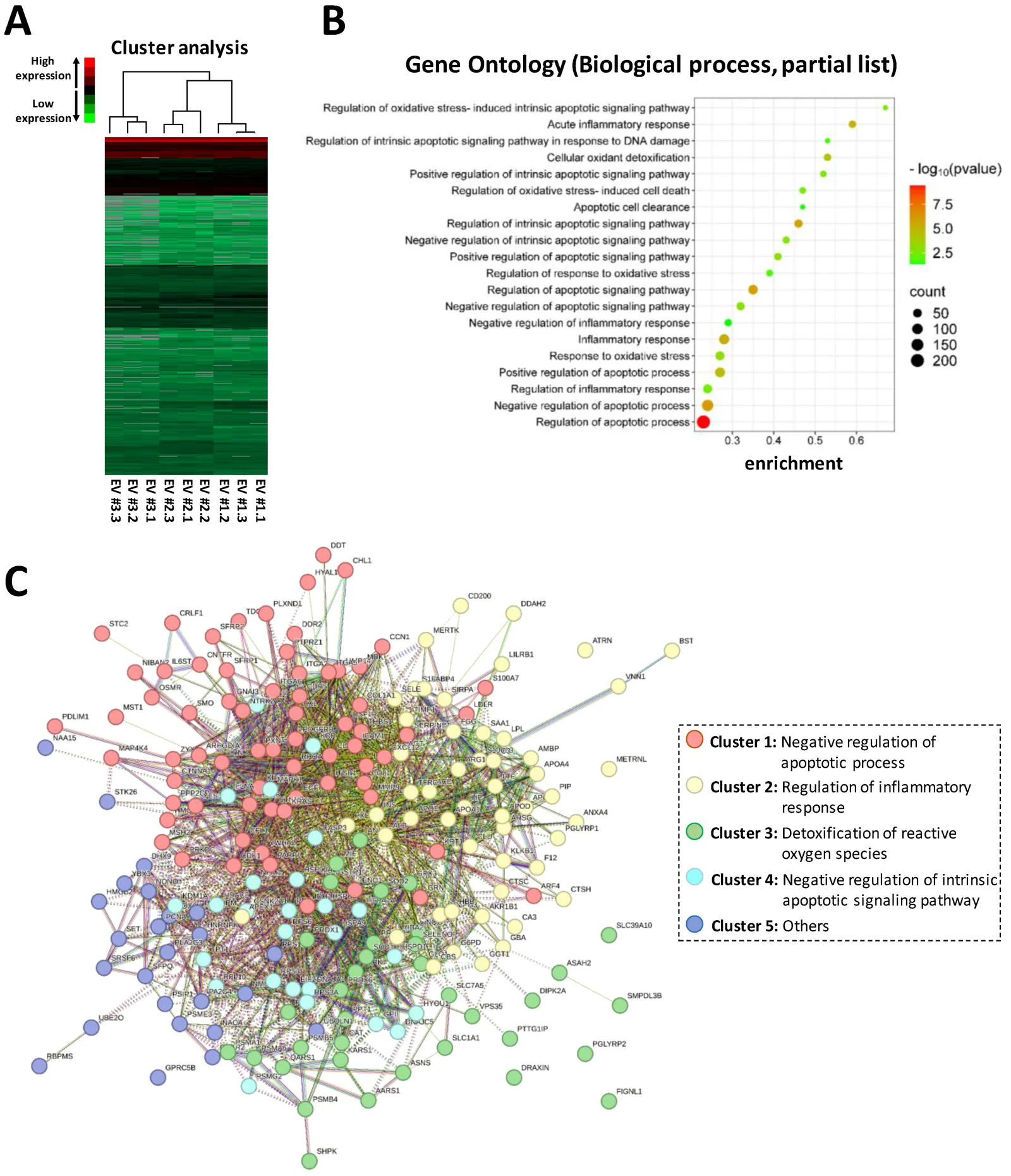
Proteomic profiling of hiPSC-EV. (A) Hierarchical clustering heatmap of proteins identified in hiPSC-EV, with red indicating high abundance and green indicating low abundance. (B) Gene ontology (GO) enrichment analysis of biological processes associated with EV proteins, showing a representative subset of significantly enriched terms. (C) Protein-protein interaction (PPI) network of EV proteins. Nodes represent proteins, and edges denote known or predicted interactions. Proteins are grouped into functionally annotated, color-coded clusters.

**Figure 3:**
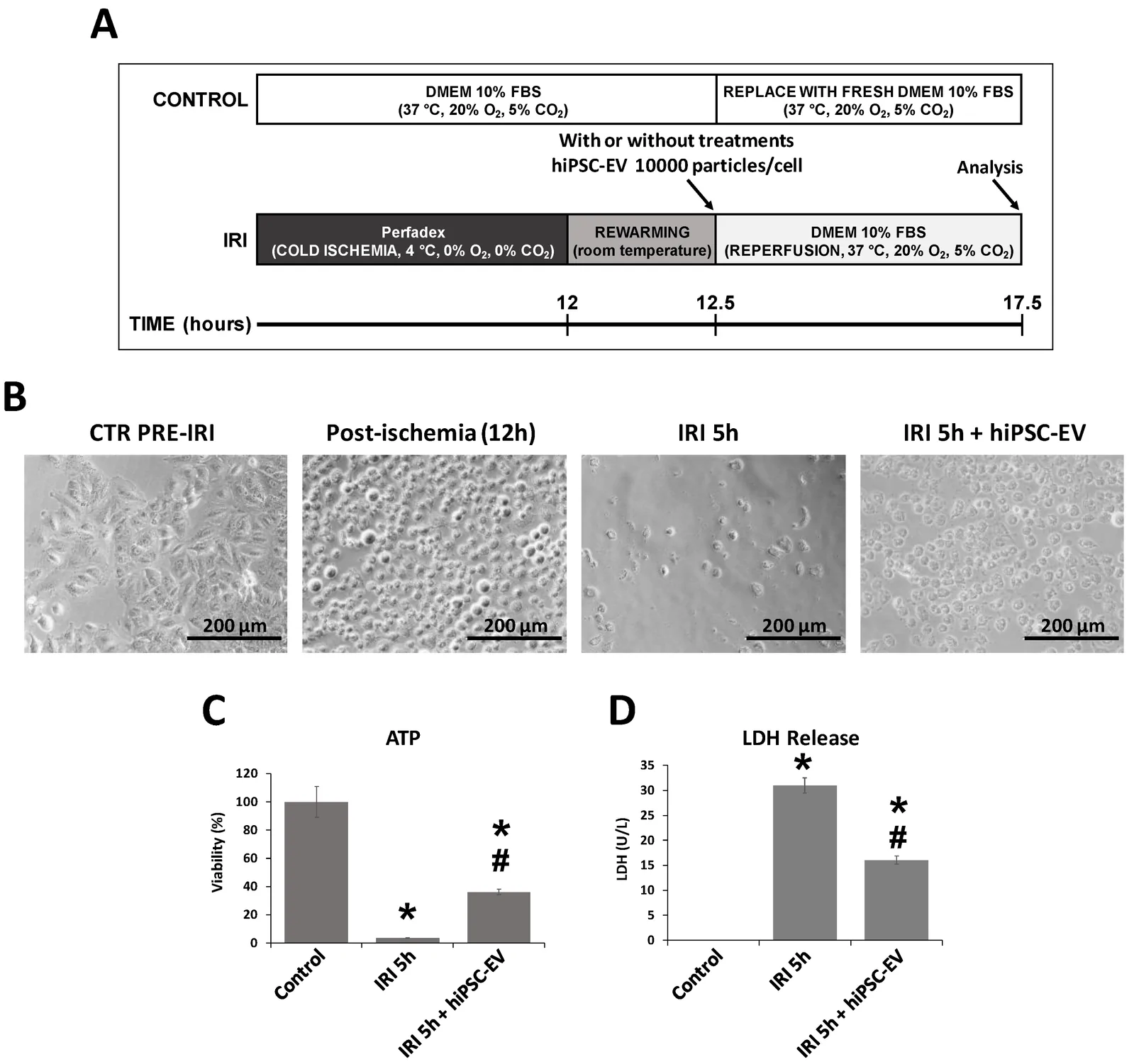
IRI model of A549 cells and protective effects mediated by hiPSC-EV treatment. (A) Schematic representation of the experimental design. (B) Representative bright-field images of A549 cell morphology at different stages: control (CTR), after 12 hours of cold ischemia (post-ischemia 12h), after 5 hours of reperfusion without hiPSC-EV (IRI 5h), and after 5 hours of reperfusion with hiPSC-EV (IRI 5h + hiPSC-EV). (C) Cell viability assessed by ATP content measurement. (D) Cell death quantified by lactate dehydrogenase (LDH) release. Data are presented as mean ± SD from at least three independent experiments. *p < 0.05 versus control; ^#^*p < 0.05 versus* IRI 5h without hiPSC-EV.

**Figure 4:**
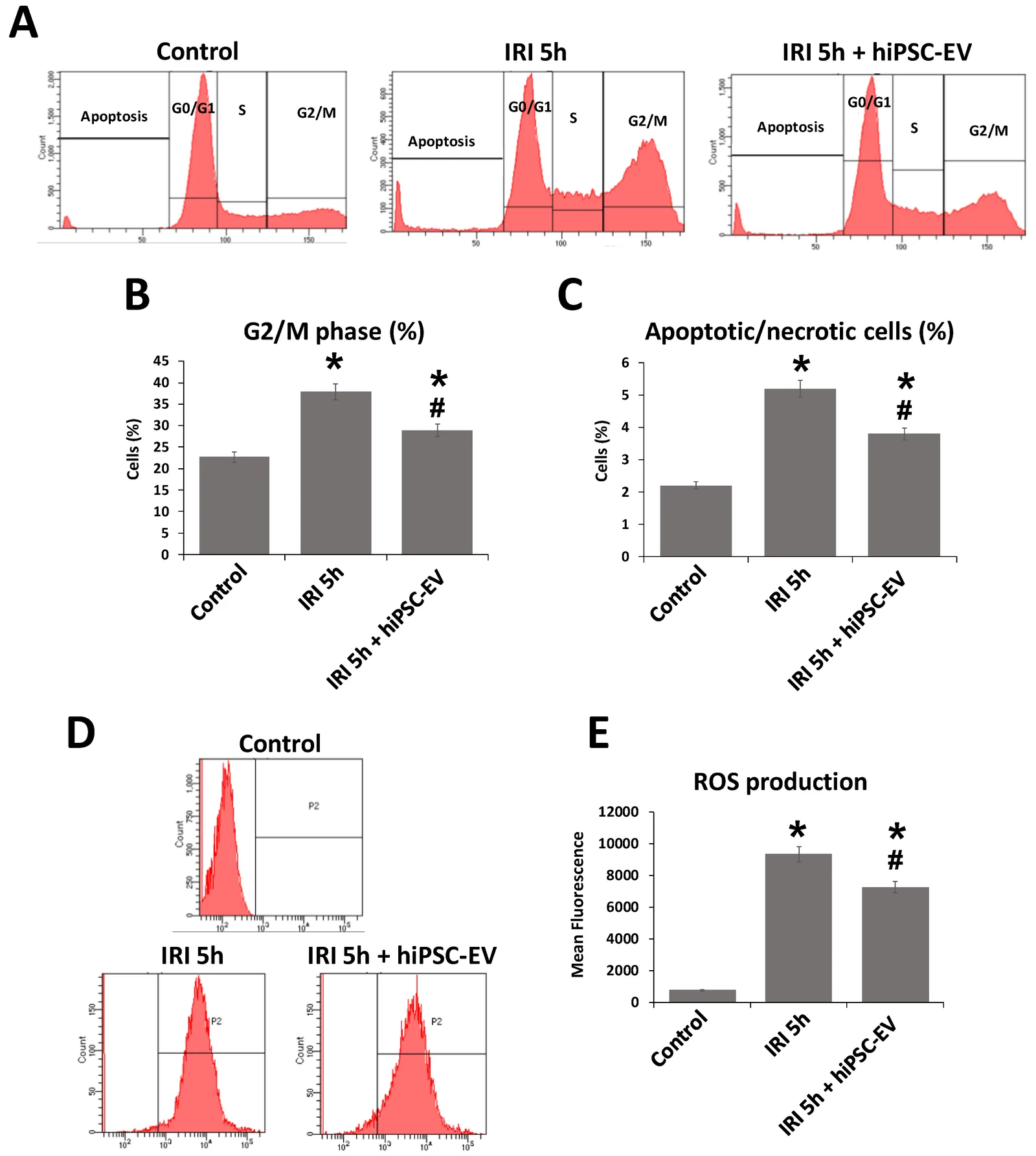
Effects of hiPSC-EV treatment on cell-cycle progression and ROS production in A549 cells subjected to IRI. (A) Representative flow cytometry histograms of DNA content illustrating cell-cycle distribution in control cells after IRI 5h and IRI 5h with hiPSC-EV. (B) Percentage quantification of cells in G2/M phase. (C) Percentage quantification of apoptotic/necrotic cells (sub-G0/G1). (D) Representative flow cytometry histograms of intracellular ROS levels measured by DCF fluorescence. The P2 gate denotes ROS-positive cells in control, IRI 5h, and IRI 5h with hiPSC-EV groups. (E) Mean fluorescence intensity of DCF, indicating ROS production. Data are presented as mean ± SD from at least three independent experiments. *p < 0.05 versus control, ^#^*p < 0.05 versus* IRI 5h without hiPSC-EV treatment.

**Figure 5:**
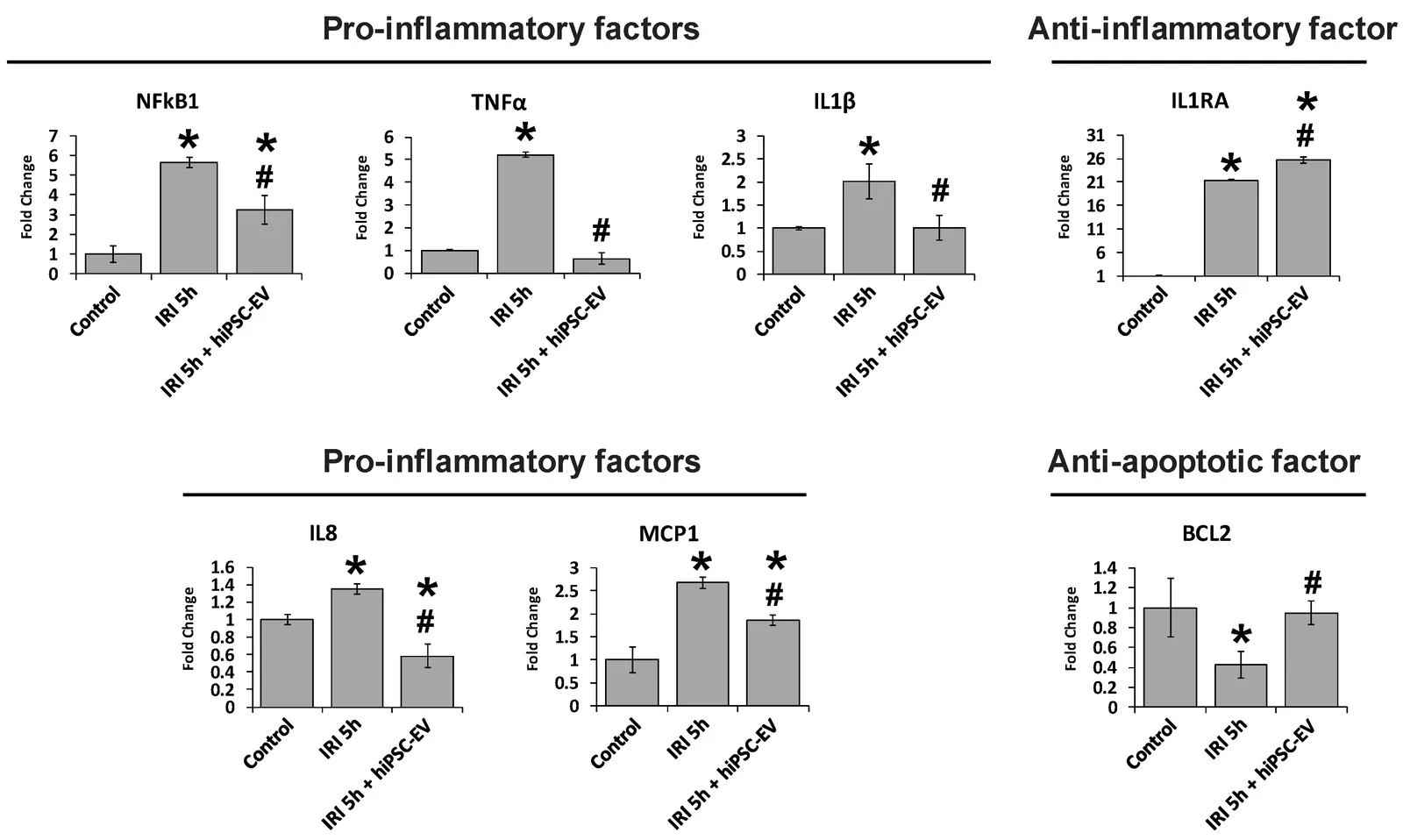
hiPSC-EV treatment modulates the expression of inflammatory and apoptotic mediators in A549 cells after IRI. Transcript levels of pro-inflammatory factors (NFkB1, TNFα, IL1β, IL8, MCP1), the anti-inflammatory mediator IL1RA, and the anti-apoptotic gene BCL2 were normalized to GAPDH and are presented as fold change relative to control. Data represent mean ± SD of three independent experiments. *p < 0.05 *versus* control; ^#^p < 0.05 *versus* IRI 5h without hiPSC-EV.

**Figure 6:**
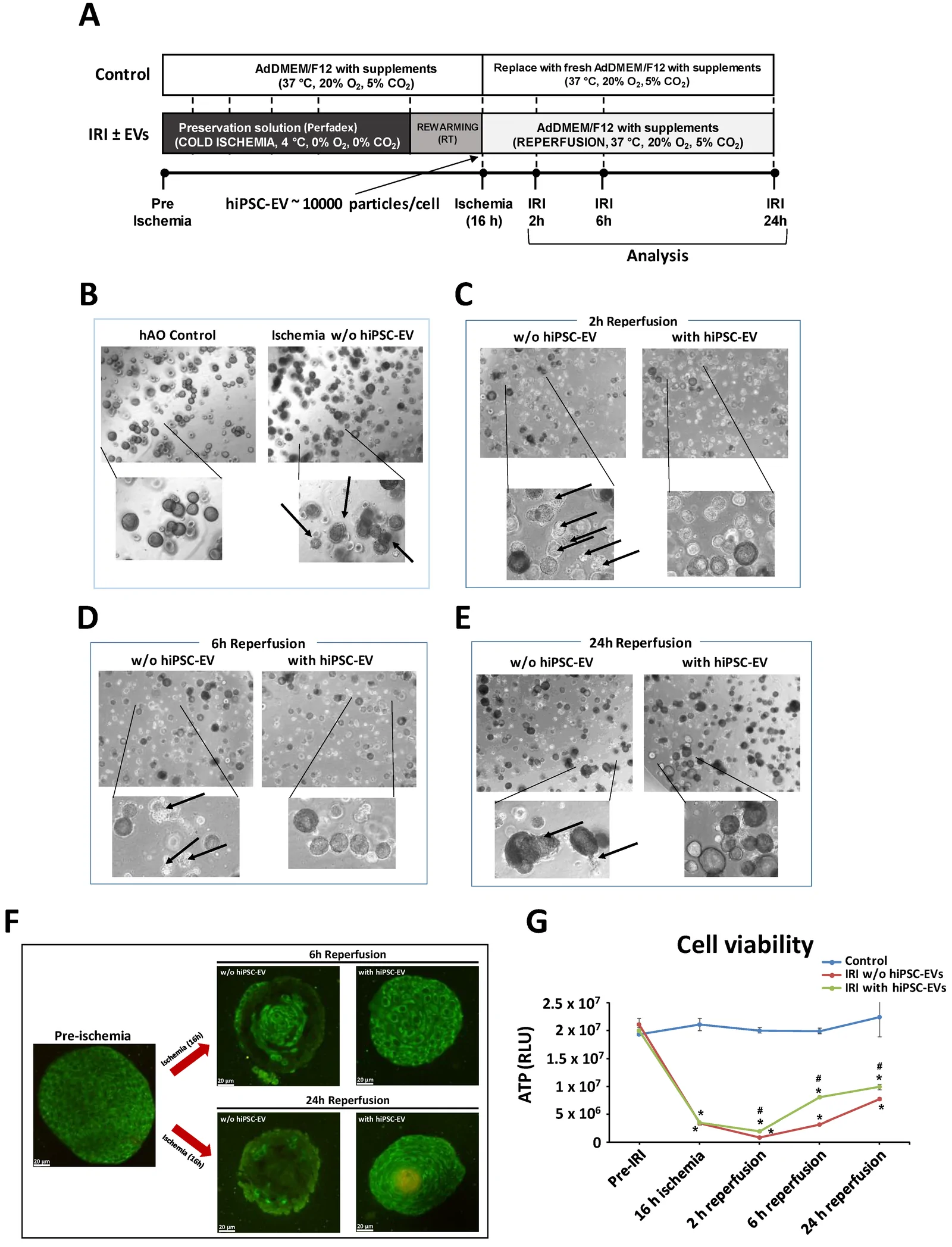
Protective effects of hiPSC-EV treatment on structural integrity and viability of hAO during IRI. (A) Experimental timeline. (B-E) Representative bright-field images (4x magnifications, EVOS microscope) of hAO pre-ischemia, post-ischemia (16 hours) and different reperfusion phases, show onset of cellular degeneration (black arrows). The bottom row of each image highlights morphological damage. (F) Label-free imaging of hAO pre-ischemia and different reperfusion phases (6 and 24 hours) performed using multiphoton microscopy. (G) hAO viability assessed by cellular ATP-dependent luminescence (RLU) at each time point. Data are mean ± SD (n = 3). Samples without hiPSC-EV treatment: w/o hiPSC-EV. Samples with hiPSC-EV treatment: with hiPSC-EV. *p < 0.05 *versus* pre-ischemia (control); ^#^p < 0.05 *versus* IRI without hiPSC-EV.

**Figure 7:**
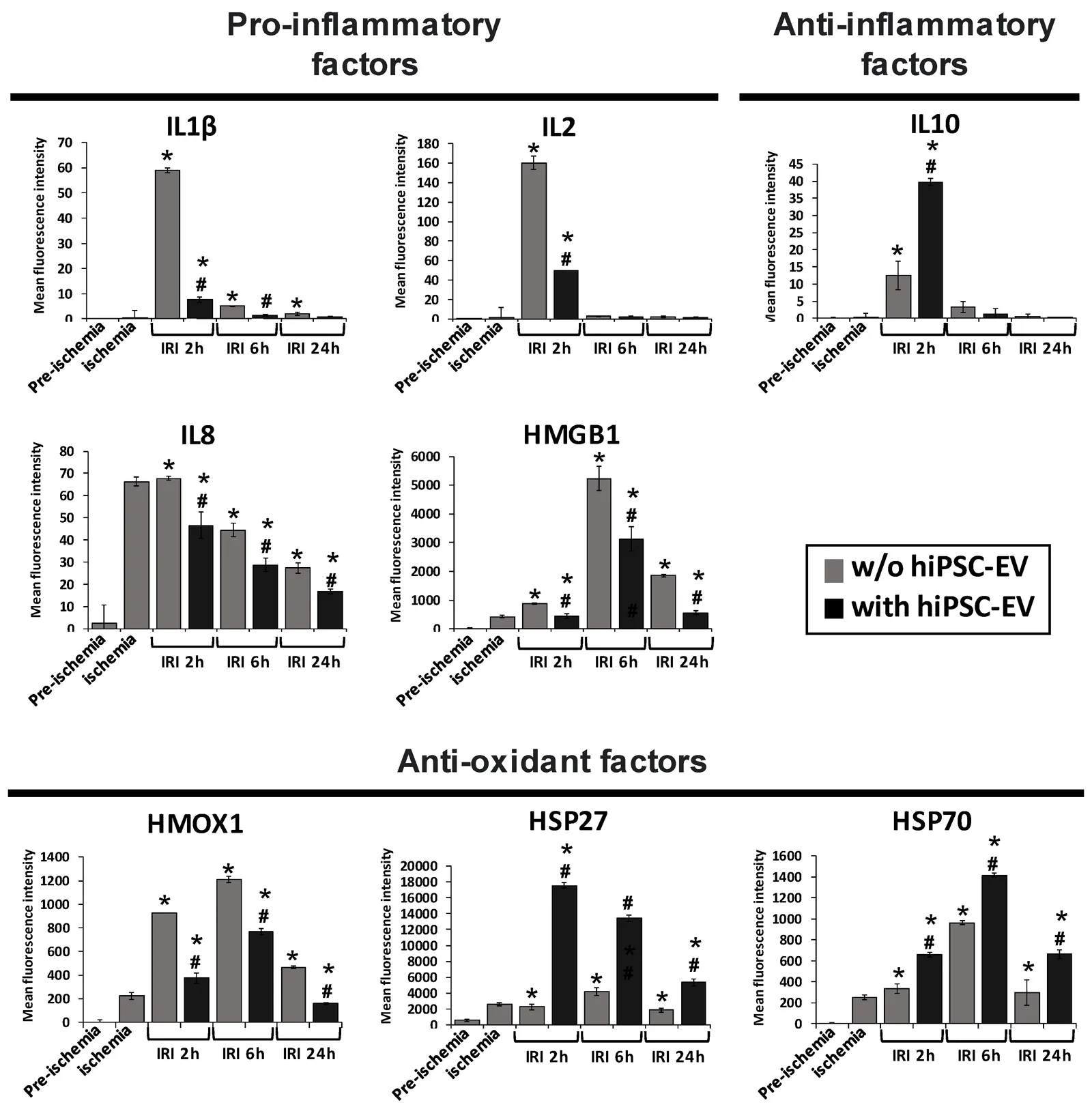
hiPSC-EV administration modulates protein expression of inflammatory and anti-oxidant mediators in hAO after IRI. Conditioned medium was collected at each different time point, and protein levels of pro-inflammatory factors (IL1β, IL2, IL8, HMGB1), anti-inflammatory cytokine IL10, and anti-oxidant regulators (HMOX1, HSP27 and HSP70) were quantified by Luminex technology. Data are mean ± SD (n = 3); *p < 0.05 versus control; *^#^p < 0.05 versus* IRI without hiPSC-EV.

**Figure 8:**
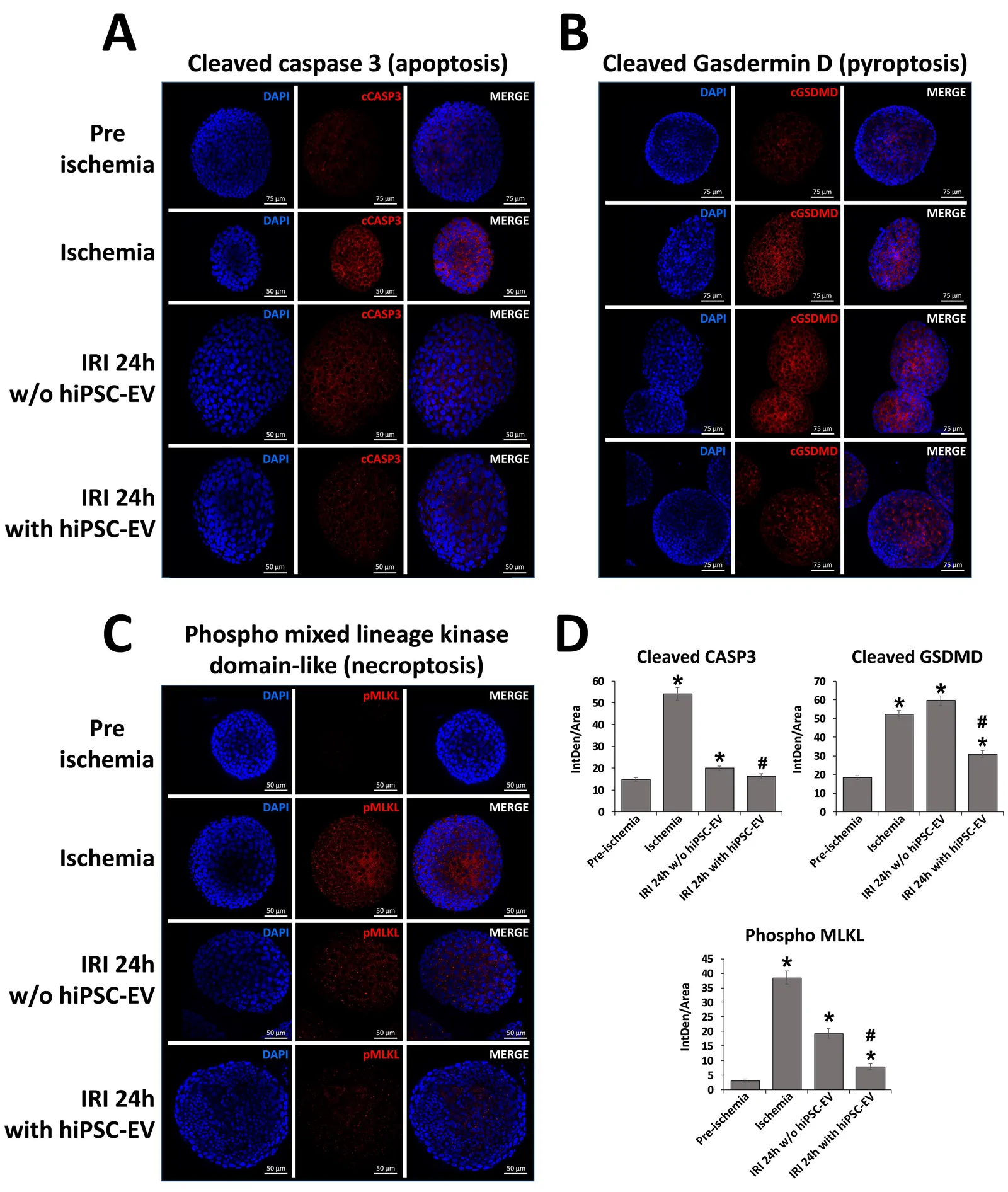
hiPSC-EV administration reduces the activation of apoptotic, pyroptotic, and necroptotic pathways in hAO post-IRI 24h. Sections were immunostained with DAPI (blue) and antibodies against (A) cleaved CASP3 (apoptosis, red), (B) cleaved GSDMD (pyroptosis, red), (C) phospho MLKL (necroptosis, red). Confocal representative images show pre-ischemia (control), post-ischemia 16h, IRI 24h without hiPSC-EV, IRI 24h with hiPSC-EV. (D) Quantification of marker intensity by integrated density (IntDen) normalized to the organoid area. Data are mean ± SD from three independent experiments. *p < 0.05 *versus* pre-ischemia (control); ^#^p < 0.05 *versus* 24h reperfusion without EV.

**Table 1:** Body weight (BW) measurements performed on animals (n=3 for each hiPSC-EV batch) that received hiPSC-EV administration (n=3 hiPSC-EV batches).

hiPSC-EV batch 1							
**mouse ID**	**BW (g)**						
Day	2	18	30	48	58	69	89
989	21.9	25.6	26.5	27.5	27.4	27.4	29.0
990	24.8	28.2	28.5	30.3	29.6	30.0	31.2
991	27.9	29.8	30.5	31.3	32.0	32.2	33.3
Mean	24.9	27.9	28.5	29.6	29.7	29.9	31.2
SD	3.0	2.1	2.0	1.9	2.3	2.4	2.2

**hiPSC-EV batch 2**							
**mouse ID**	**BW (g)**						
Day	2	18	30	48	58	69	89
992	21.3	23.5	24.9	26.7	27.2	27.1	29.3
993	21.1	25.7	26.8	27.8	27.1	27.4	29.2
994	25.8	27.5	27.7	29.8	28.8	29.8	30.3
Mean	22.7	25.6	26.5	28.1	27.7	28.1	29.6
SD	2.7	2.0	1.4	1.6	1.0	1.5	0.6

**hiPSC-EV batch 3**							
**mouse ID**	**BW (g)**						
Day	2	18	30	48	58	69	89
995	28.5	30.0	32.4	32.1	31.8	32.6	33.7
996	26.9	31.1	32.2	30.6	29.2	30.7	32.6
997	27.5	28.8	31.2	31.8	30.8	30.7	33.3
Mean	27.6	30.0	31.9	31.5	30.6	31.3	33.2
SD	0.8	1.2	0.6	0.8	1.3	1.1	0.6


## Data Availability

Proteomic data presented in the study are deposited in the ProteomeXchange repository with the dataset identifier PXD068292. Other original data presented in the study are openly available at the following link: https://osf.io/qh6cf/overview?view_only=83a037cb80104875a557c7934bfd8a54.

## Abbreviations

2D: Two-dimensional
3D: Three-dimensional
AcTub: Acetylated α-tubulin
BCL2: B-cell lymphoma 2
BME 2: Basement Membrane Extract Type 2
BSA: Bovine serum albumin
CAT: Catalase
cCASP3: Cleaved caspase 3
cGSDMD: Cleaved gasdermin D
CLAD: Chronic lung allograft dysfunction
DAPI: 4′,6-diamidino-2-phenylindole dihydrochloride
DCFH-DA: 2′,7′-Dichlorodihydrofluorescein diacetate
DIA: Data-independent acquisition
DPBS: Dulbecco's phosphate-buffered saline
EV: Extracellular vesicles
EVLP: *Ex vivo* lung perfusion
FASP: Filter-aided sample preparation
GAPDH: Glyceraldehyde 3-phosphate dehydrogenase
GO: Gene ontology
GRN: Granulin
hAO: Human airway organoid
hiPSC-EV: Extracellular vesicles derived from human induced pluripotent stem cells
hiPSCs: Human induced pluripotent stem cells
HLA: Human leukocyte antigen
HMGB1: High mobility group box 1
HMOX1: Heme oxygenase 1
HSP27: Heat shock protein 27
HSP70: Heat shock protein 70
IL1β: Interleukin 1 beta
IL1RA: Interleukin 1 receptor antagonist
IL2: Interleukin 2
IL8: Interleukin 8
IL10: Interleukin 10
IRI: Ischemia-reperfusion injury
KRT5: Cytokeratin 5
LDH: Lactate dehydrogenase
LFQ: Label-free quantification
LTx: Lung transplantation
MAF: Musculoaponeurotic fibrosarcoma oncogene homolog
MCP1: Monocyte chemoattractant protein 1
METRNL: Meteorin-like protein
miRNAs: MicroRNAs
MS: Mass spectrometry
MSC: Mesenchymal stromal/stem cell
MSC-EV: Extracellular vesicles derived from mesenchymal stromal/stem cell
MUC1: Mucin 1
NFkB1: Nuclear factor kappa B subunit 1
NG2: Chondroitin Sulfate Proteoglycan 4
NRF2: Nuclear factor erythroid 2-related factor 2
NTA: Nanoparticle tracking analysis
PGD: Primary graft dysfunction
PI: Propidium iodide
pMLKL: Phospho mixed lineage kinase domain-like protein
PPI: Protein-protein interaction
ROR1: Receptor tyrosine kinase like orphan receptor 1
ROS: Reactive oxygen species
RT: Room temperature
SCGB1A1: Secretoglobin
SEM: Scanning electron microscopy
SOD1: Superoxide dismutase 1
SOD2: Superoxide dismutase 2
SSEA-4: Stage-specific embryonic antigen-4
TNFα: Tumor necrosis factor alpha
