# Supplementary material for: Extracellular Vesicles from Human Induced Pluripotent Stem Cells Reduce Lung Ischemia-Reperfusion Injury via Cytoprotective Mechanisms in both 2D Cultures and a Human Airway Organoid Model
Source: Int J Biol Sci. 2026 Oct 2;22(15):8571–90. doi: 10.7150/ijbs.134686 (PMC13685754; doi:10.7150/ijbs.134686)
Supplement: Supplementary file 1 [file ijbsv22p8571s1.pdf]

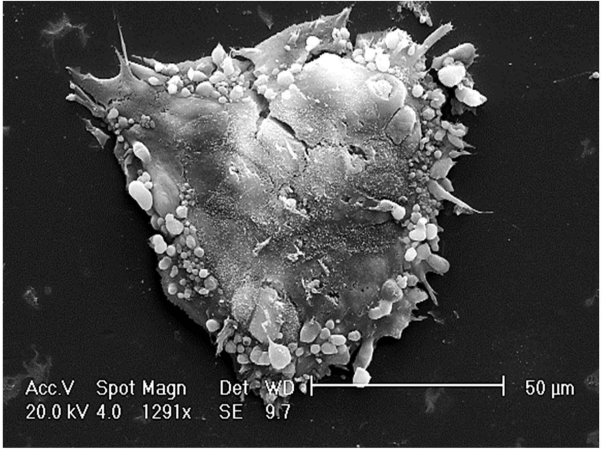
Acc.V Spot Magn Det WD
20.0 kV 4.0 1291x SE 9.7
50 µm

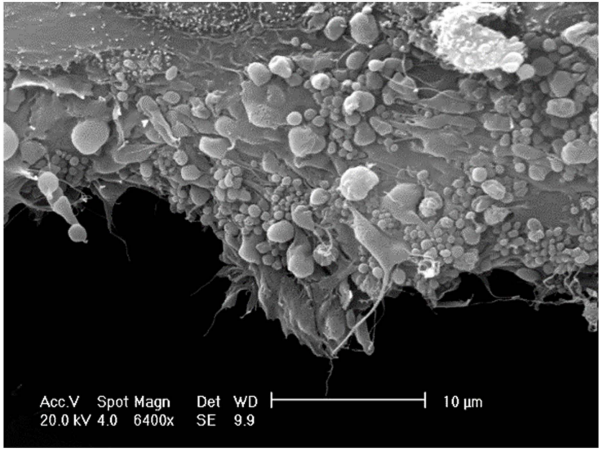
Acc.V Spot Magn Det WD
20.0 kV 4.0 6400x SE 9.9
10 µm

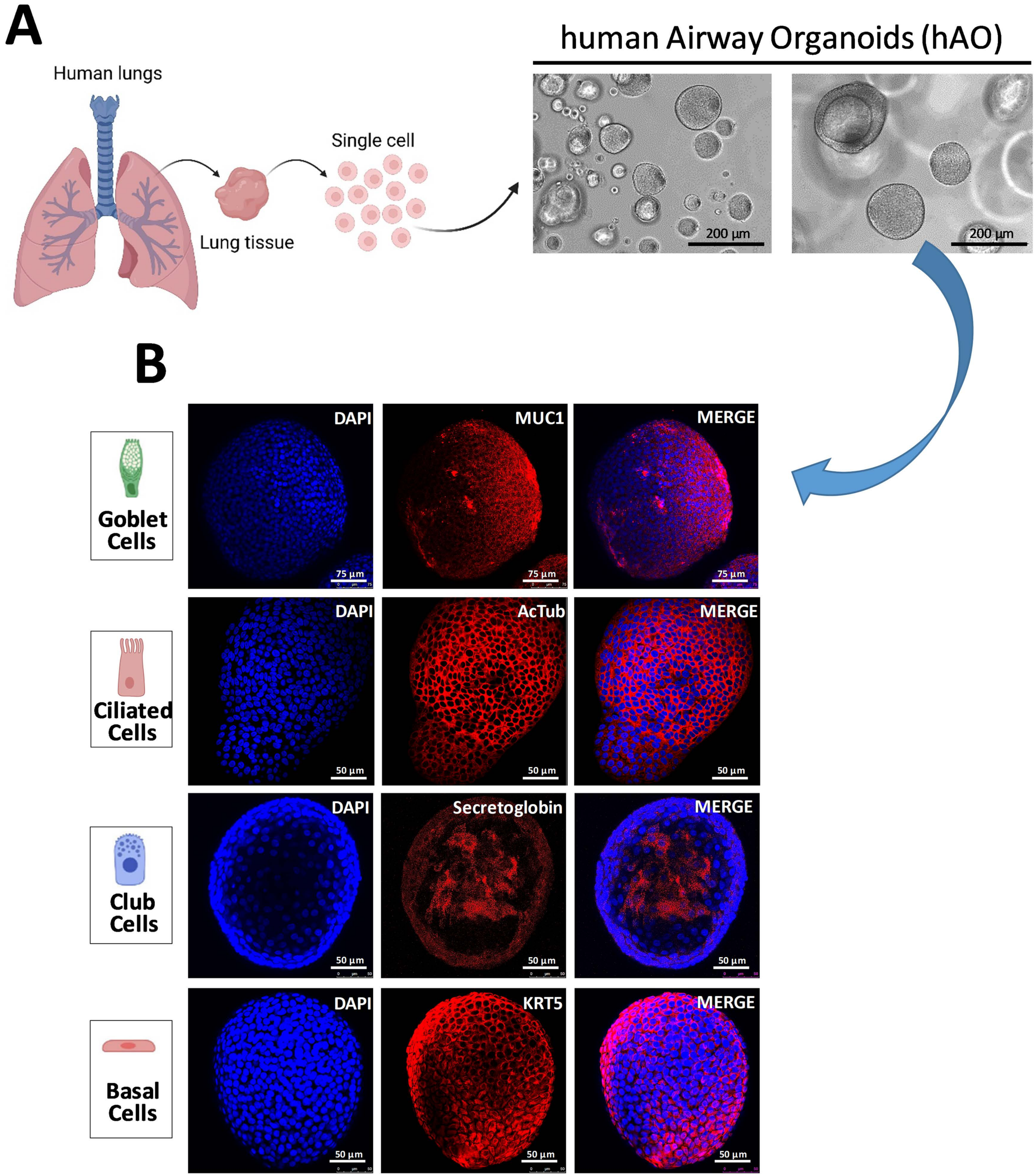
A
Human lungs
Lung tissue
Single cell
human Airway Organoids (hAO)
200 µm
200 µm
B
Goblet Cells
DAPI
MUC1
MERGE
75 µm
75 µm
75 µm
Ciliated Cells
DAPI
AcTub
MERGE
50 µm
50 µm
50 µm
Club Cells
DAPI
Secretoglobin
MERGE
50 µm
50 µm
50 µm
Basal Cells
DAPI
KRT5
MERGE
50 µm
50 µm
50 µm

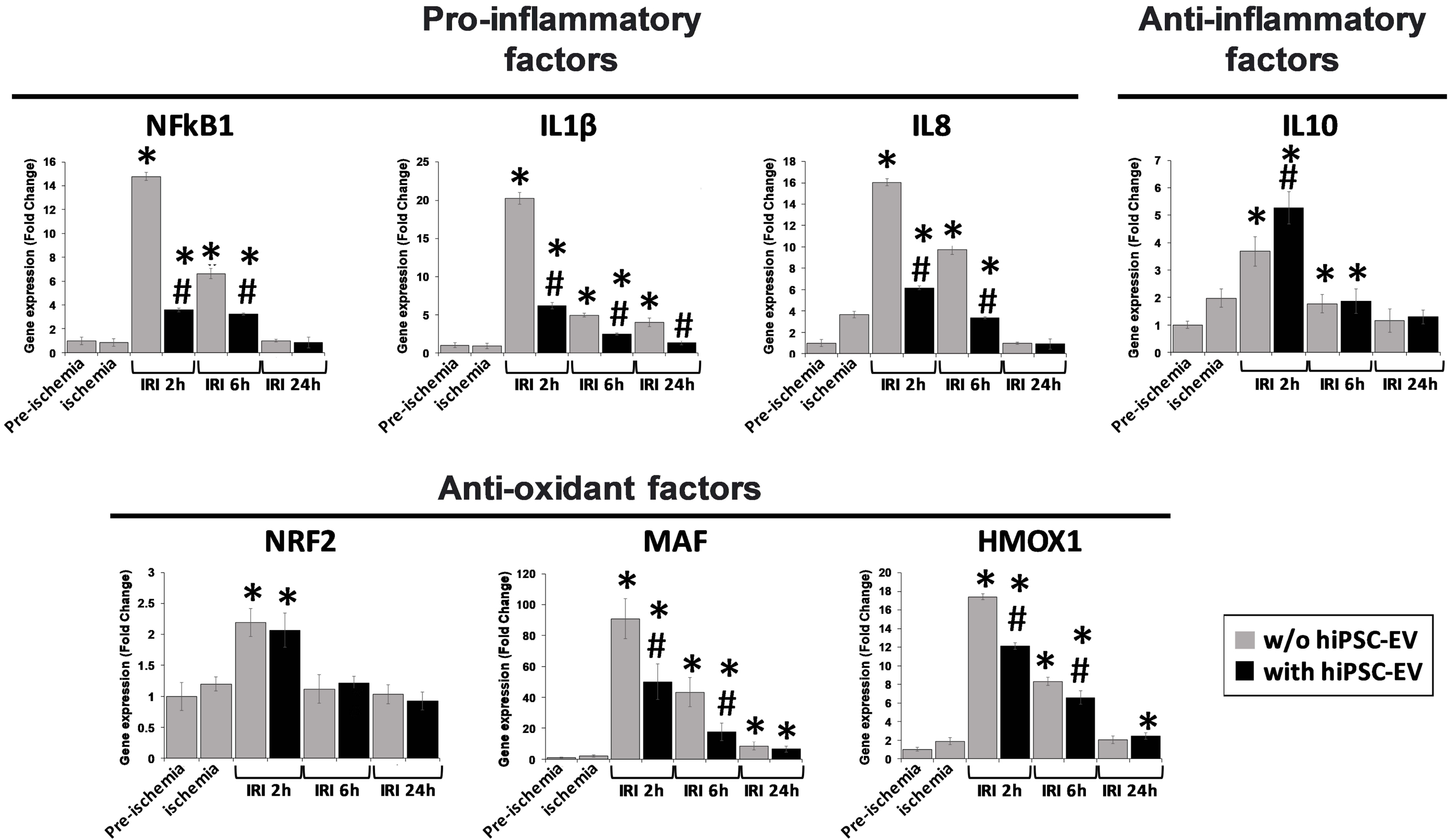

Pro-inflammatory factors
Anti-inflammatory factors
NFkB1
IL1β
IL8
IL10
Anti-oxidant factors
NRF2
MAF
HMOX1
Gene expression (Fold Change)
Pre-ischemia
ischemia
IRI 2h
IRI 6h
IRI 24h
w/o hiPSC-EV
with hiPSC-EV
